# Correction: Plumbagin protects mice from lethal Sepsis by modulating immunometabolism upstream of PKM2

**DOI:** 10.1186/s10020-025-01329-9

**Published:** 2025-07-25

**Authors:** Zhaoxia Zhang, Wenjun Deng, Rui Kang, Min Xie, Timothy Billiar, Haichao Wang, Lizhi Cao, Daolin Tang

**Affiliations:** 1 Department of Pediatrics, The Second Affiliated Hospital of Jinan University, Shenzhen People’s Hospital, Shenzhen, Guangdong People’s Republic of China; 2 Department of Pediatrics, Xiangya Hospital, Central South University, Changsha, Hunan People’s Republic of China; 3 Department of Surgery, University of Pittsburgh, Pittsburgh, Pennsylvania, USA; 4 Laboratory of Emergency Medicine, The Feinstein Institute for Medical Research, Manhasset, NY USA; 5 Center for DAMP Biology, The Third Affiliated Hospital of Guangzhou Medical University, Guangzhou, Guangdong People’s Republic of China

**Correction: Mol Med 22, 162–172 (2016)**

**https://doi.org/10.2119/molmed.2015.00250**

Following publication of the original article (Zhang et al. 2016), the wrong figure appeared as Fig. 2.; the figure should have appeared as shown below.

**Fig. 2:**
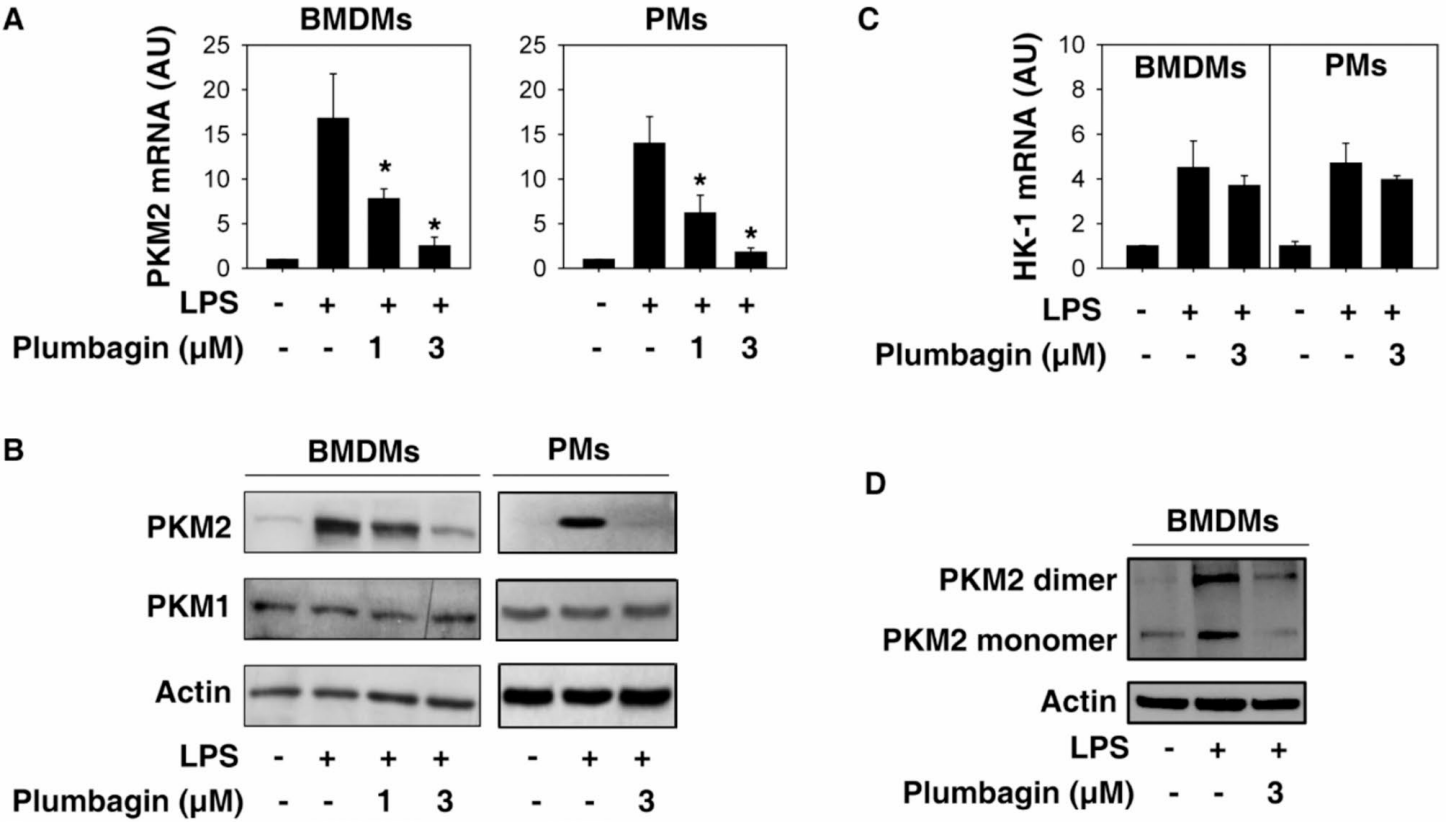
Plumbagin inhibits PKM2 expression in activated macrophages. (**A**–**C**) Indicated macrophages were treated with lipopolysaccharide (LPS) (100 ng/mL) in the absence or presence of plumbagin for 24 h. The mRNA or protein levels of PKM2 (**A**-**B**) or HK-1 (**C**) were assayed using Q-PCR and western blot, respectively (*n* = 3, *, *p* < 0.05 versus LPS group). (**D**) Native gel electrophoresis was performed using whole-cell extracts from BMDMs after treatment with LPS (100 ng/mL) and/or plumbagin (3 µmol/L) for 24 h
